# viruSITE—integrated database for viral genomics

**DOI:** 10.1093/database/baw162

**Published:** 2016-12-26

**Authors:** Matej Stano, Gabor Beke, Lubos Klucar

**Affiliations:** Laboratory of Bioinformatics, Institute of Molecular Biology, Slovak Academy of Sciences, Bratislava, Slovakia

## Abstract

Viruses are the most abundant biological entities and the reservoir of most of the genetic diversity in the Earth's biosphere. Viral genomes are very diverse, generally short in length and compared to other organisms carry only few genes. viruSITE is a novel database which brings together high-value information compiled from various resources. viruSITE covers the whole universe of viruses and focuses on viral genomes, genes and proteins. The database contains information on virus taxonomy, host range, genome features, sequential relatedness as well as the properties and functions of viral genes and proteins. All entries in the database are linked to numerous information resources. The above-mentioned features make viruSITE a comprehensive knowledge hub in the field of viral genomics.

The web interface of the database was designed so as to offer an easy-to-navigate, intuitive and user-friendly environment. It provides sophisticated text searching and a taxonomy-based browsing system. viruSITE also allows for an alternative approach based on sequence search. A proprietary genome browser generates a graphical representation of viral genomes. In addition to retrieving and visualising data, users can perform comparative genomics analyses using a variety of tools.

**Database URL**: http://www.virusite.org/

## Introduction

Viruses are the simplest biological forms bordering on the edge between living and non-living. Nevertheless, their genomes are more diverse than any other forms of life. Viral genomes vary in terms of their length (from 1.7 kb of circoviruses to 2500 kb of *Pandoravirus salinus*), composition (ssRNA, dsRNA, ssDNA and dsDNA), topology (linear, circular) and consist of a single strand or multiple segments of nucleic acid (1). A common feature of all viral genomes is their small size (mostly <50 kb) and high density of gene coding regions (mostly >90%).

Genomes of viruses (bacteriophages MS2 and phiX174) were the first to be sequenced (2, 3). At present, there are thousands of known viral genome sequences. The NCBI Viral Genomes Resource contains over 5600 complete reference viral genomes (http://www.ncbi.nlm.nih.gov/genomes/Genom esGroup.cgi?taxid=10239). In total, >2 million sequences of viral origin are deposited in INSDC databases (http://www.ncbi.nlm.nih.gov/nuccore/?term= txid10239[Organism:exp]). Next-generation sequencing technology brought a rapid increase in sequenced genomes and established new challenges associated with annotating and maintaining viral genomes (4).

There are various public databases which store and distribute information regarding viral genome sequences, annotation of genes and proteins, information on viral structure, biology, pathology and taxonomy. Primary sequence databases, INSDC databases (GenBank/ENA/DDBJ) (5) and Uniprot (6) constitute the main repositories of sequence data concerning nucleotide and protein levels. ViralZone is a curated virus knowledge base which focuses on molecular biology of viruses, virion structure, genome, replication cycle, host range, geographical distribution and epidemiology (7). ViPR is an integrated data repository which provides tools for analysis of human pathogenic viruses (8). Reports of the International Committee on the Taxonomy of Viruses (ICTV) (9) are considered both the standard and the reference for the taxonomy of viruses. The taxonomy releases are also available online (www.ictvonline.org). Furthermore, there are databases focusing on specific groups of viruses, e.g. OpenFluDB (10), Ebola Virus Knowledgebase (11), HBVdb (12) and many others.

We present here the viruSITE—an integrated database for viral genomics. The main aim of this database is to establish a general and comprehensive information resource in the field of viral genomics. viruSITE combines data and information from numerous resources and presents them in a concise and comprehensible form. It is not merely a derived repository of these data but rather a system that provides a unique approach to information retrieval, analysis and visualization. Among others, viruSITE also offers the following features:

- a versatile search engine (e.g. searching by virus name, host name, GO derived protein function or sequence similarity);
- taxonomy-based browsing of the virus collection;
- convenient searching of gene/protein homologs and virus relatives;
- schematic visualization of sequence similarity between viral genomes;
- graphical and interactive viral genome browser;
- integrated suite of tools for sequence analysis.

## Data processing and content

With the aim to create a broader annotation system for genomes, genes and proteins encompassing the whole universe of viruses, the data were collected from several resources. All genome sequences in viruSITE were extracted from all reference viral genomes deposited in the NCBI RefSeq database (records with ‘NC_’ accession prefix). The annotation acquired from the RefSeq entries was amended with the data obtained from UniProtKB, Pfam, GO, ViralZone, NCBI Taxonomy and PubMed. All the data were downloaded, parsed, pre-computed, combined and inserted into the database using in-house developed scripts. All scripts work semi-automatically, and therefore human supervision is necessary.

The database is regularly updated (at least two releases per year). Since 2013, when viruSITE was established, the total number of records has doubled. The current release (2016.2) contains records for 5633 viruses, viroids and satellites (7312 genome sequences in total), 273 366 records for genes and 269 157 records for proteins. The content of the database is available to everyone for any purpose and it is distributed under the ‘Creative Commons Attribution-Share Alike 3.0 Unported License’.

## Database construction

viruSITE is a web-based relational database utilizing MySQL. It operates on a Linux Slackware system under an Apache web server. Its web interface was developed using HTML/PHP. The viruSITE web pages make use of JavaScript/Ajax in order to enhance their dynamics and minimize data transfer. The proprietary genome browser phiGENOME, which is integrated into the viruSITE web interface framework, is built on Adobe Flash technology (13).

Apart from PHP scripts handling database queries from users, various sequence analysis and visualization tools run in the viruSITE environment as well. BLAST programs were implemented into the web interface to perform sequence similarity searches (14). Circoletto, a visualization tool based on Circos, provides a graphical display of sequence similarity on full genome scale (15, 16) and also visual overview of sequence similarity between sequentially related viral genomes. The MUSCLE program was employed for multiple sequence alignment and construction of phylogenetic trees (17). The jsPhyloSVG, JavaScript library, was used for visualising interactive vector-based phylogenetic trees via web interface (18).

In addition to software running on a local server, BLAST programs are also executed on remote NCBI servers. For protein domain identification, Pfam searches are executed on remote servers of the Wellcome Trust Sanger Institute (19). A RESTful web service was implemented for programmatic interaction between local and remote servers. viruSITE offers a simple way to identify sets of homologous genes and proteins as well as groups of related viral genomes. Sequence homology is determined by a local BLAST search. For gene and protein queries, the similarity is calculated on the fly, whereas for whole genome sequences it is pre-computed in a pairwise manner for each database release. BLAST searches are executed with default parameters, with the exception of those used to identify a virus’ relatives, where the threshold *E*-value of 1e−10 is used.

## Database interface

**Figure 1.**
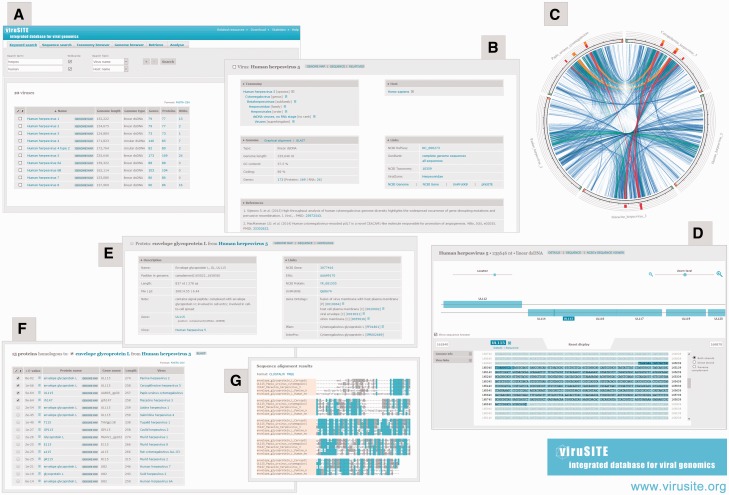
viruSITE web interface. (**A**) Keyword search—a search result for human herpes viruses. (**B**) Entry details page for human herpesvirus 5 (HHV-5). (**C**) Circoletto visualization of the sequence similarity between genomes related to HHV-5. (**D**) HHV-5 genome presented in the genome browser: visualization focused on glycoprotein L. (**E**) Entry details page for glycoprotein L from HHV-5. (**F**) List of proteins homologous to glycoprotein L from HHV-5. (**G**) Multiple sequence alignment of selected glycoprotein L sequences from five different herpes viruses.

The viruSITE database is accessed via a web-based interface divided into six main sections: *Keyword search, Sequence search, Taxonomy browser, Genome browser, Retrieve and Analyse* (Figure 1).

*Keyword search* provides a straightforward way to query the database. Search terms are looked up in conjunction with the following fields: Virus name, Taxonomy ID, Host name, Host group (terms used by the NCBI Viral Genome Resource), Genome type, Genome length and Genome RefSeq ID, Gene/protein name, Entrez Gene ID, Entrez Protein ID, UniProt ID, GO ID, GO term and Pfam ID. Up to three search terms/fields can be combined in order to refine the search. When utilizing one of the ‘name’ fields, a wildcard character can be used as a suffix attached to a specific search term. Official scientific names (e.g. Variola virus, *Homo sapiens*) and common names (e.g. smallpox virus, human) are equally accepted.

*Sequence search* allows scanning a sequence of interest against all sequences of viral genomes and proteins in the database. Local BLAST search is employed to determine sequence similarity.

In the *Taxonomy Browser* section, users can browse through the list of all viral species and higher taxonomic ranks deposited in the viruSITE database. Names of taxons are sorted alphabetically, and browsing through this list is facilitated by an Ajax auto-complete search script. In addition, this section includes a drop-down taxonomic tree that enables hierarchical browsing throughout the viral taxonomy.

The search/browse output is displayed in a tabular form and can be directly downloaded in a CSV format. Sequences can be downloaded in FASTA format. The data in the table are sorted by clicking the column title. Users can also change the number of displayed results per page. Selecting individual checkboxes located in the first column of the results table enables retrieval and analysis of the corresponding sequences (see the *Retrieve* and *Analyse* sections below).

After clicking on an entry name, the entry details page is displayed. The virus details page contains information on virus nomenclature (official and alternative names), taxonomy, virus hosts, genome properties (type, length, number of segments, GC content, coding percentage, number of encoded genes, proteins and RNAs) and links to other online databases and literature references. The gene/protein details page contains names, genomic position, length, molecular weight and pI, gene ontologies, protein domains and numerous links to other online databases and literature references. Furthermore, each entry’s details page is linked to the graphical representation of a viral genome in the phiGENOME browser (*Genome Browser* section), to the sequence display in a pop-up window as well as to the homology search and visualization.

The *Genome Browser* section of the viruSITE web portal utilizes phiGENOME, the proprietary graphical genome browser. It provides a dynamic and interactive graphical representation of viral genomes. The genome browser consists of two interconnected components: (i) An Adobe Flash-based graphical applet, providing dynamic and interactive graphical display of genomes, and (ii) semi-graphical HTML-based output, providing detailed exploration of genome features on the sequence level. Both components are interconnected, thus any action performed in one of them (e.g. a change in zoom, position or gene selection) is instantly reflected in the other one.

In the *Retrieve* section, users can view and download selected entries from the Search/Browse output table in a CSV format and sequences in FASTA format. Furthermore, complete collections of genome, gene and protein sequences deposited in the viruSITE database are available for retrieval on the *Download* page.

The Analyse page provides a suite of tools for general sequence analyses of selected sequences:

- *Similarity search*—BLAST programs are employed for a sequence similarity search on the nucleotide and amino acid level. A search may be executed either against a complete non-redundant database (executed on a remote NCBI server) or against a non-redundant database of viral sequences (on a local server). Circoletto provides a graphical visualization of the sequence similarity (calculated by BLAST) between two or more viral genomes.
- *Protein domain search*—a search against the Pfam HMM library is used for the identification of protein domains. Analysis results are in tabular and semi-graphical form and are linked to the complete protein domain description on the Pfam web site.
- *Multiple sequence alignment*—the MUSCLE program is used for multiple sequence alignments. These alignments are formatted in colours according to the sequence similarities and complemented with a phylogenetic tree (jsPhyloSVG), showing the evolutionary distance between sequences.

Users may also utilize these tools for an analysis of any nucleotide and amino acid sequences inserted into the relevant text area.

All sections of the viruSITE web portal are interconnected and form a user-friendly environment which allows swift retrieval of high-value information. With the aim to introduce the viruSITE functionalities to users, the Help page has also been made available. It contains brief video tutorials, showing the basics for each section described above.

## Discussion and conclusion

There are numerous public databases which store information on viral genome sequences, genes and proteins, however none of them provides the same information and functionalities as viruSITE. Most of databases are focused only on specific viruses (e.g. influenza virus or HIV) or groups of viruses (e.g. hepatitis viruses or RNA viruses) (20), while viruSITE is a general resource for all virus taxa. NCBI’s Viral Genome Resource is a complete repository for all reference viral genomes (4), and it provides curated sequence data and annotation for the complete viral genome sequences deposited in the RefSeq database. However, the user interface offers limited options for searching this database. For example, it is not possible to search protein entries directly. VirGen (A Comprehensive Viral Genome Resource) was developed with the aim to serve as an annotated and curated database for all complete viral genomes, but this project has been idle for several years (21). ViralZone is another comprehensive, visually attractive and virus-oriented information resource (7). While ViralZone contains examples from all known groups of viruses, there are relatively few reference species within each group (around 10% of the viruses covered by viruSITE). Users can easily retrieve and analyse protein sequences, but are limited to those which are deposited in UniProtKB/Swiss-Prot, and the information on viral genomes in ViralZone is covered only marginally. There are also several predominantly single-purpose analysis tools available. For example, Base-By-Base is available for the whole genome alignment (22) while ViroBLAST is available for the homology search among different datasets (23). However, some of the tools available in the past are not functional anymore, e.g. GIB-V (24) or Alvira (25).

The viruSITE database described here is an integrated database for general viral genomics. The database is currently holding entries for 5633 viruses, viroids and satellites. The primary aim of viruSITE is to merge virus-oriented information from existing relevant sources and bring it to users in a concise and comprehensible form. A majority of this information is automatically extracted from the records found in reference databases (RefSeq, UniProtKB, Pfam, GO, ViralZone, NCBI Taxonomy and PubMed). However, it is not merely a derivative of existing databases. The viruSITE content is manually and semi-automatically enhanced with the data that are not explicitly present (or are incomplete) in the records of reference databases, e.g. the names of genome segments (for viruses with a segmented genome and originated from provisional RefSeq records), the percentage of coding sequences in genomes or the isoelectric point for proteins. The virus-to-host assignment has been improved by combining RefSeq and UniProt entries. The unique data stored in the database are the mutual sequence similarity scores, defining groups of viral genomes which are related on the sequence level. These are calculated in a pairwise manner for each database release (see the ‘Database construction’ section for more details). viruSITE provides a user-friendly web interface coupled with powerful tools for searching, browsing, analysis and visualization. The search form provides versatile options in terms of retrieving complex information, e.g. searching for viruses according to the host organism, taxonomical classification and genome properties. Furthermore, users can search for viral proteins according to the presence of a specific Pfam domain or on the basis of the gene ontology classification. A combination of multiple search terms refines the search procedure and offers filtering of the data relevant from a biological point of view. Particularly useful is the search based on sequence similarity which allows a quick finding of groups of homologous genes, proteins and related viral genomes. All search results pages provide export features in different formats. The graphics generated by the Circoletto/Circos bundle provides a schematic representation of sequence similarity at the level of entire viral genomes. Another available visualization utility is phiGENOME—a genome browser providing a dynamic and responsive representation of a genome organization. A set of analysis tools for comparative genomics was incorporated into the user interface. All these features make viruSITE a unique and complex knowledge resource in the field of viral genomics. viruSITE is hosted on the server of the Institute of Molecular Biology, Slovak Academy of Sciences and is freely accessible via the web portal virusite.org.

## Future perspectives

The viruSITE database has been in continuous development since 2013. In the future, the database system as well as the user interface will be continuously maintained and upgraded. The database content will be updated as well, and new entries will be added to the database as novel viral genomes are being published in the RefSeq database. In the near future, we also plan to integrate other tools for comparative genomics, e.g. genome-scale sequence alignment software. Moreover, there are plans to upgrade the Circos-based genome visualization in order to make it more interactive and customizable. Finally, additional analysis tools will be implemented as per requirements of the viruSITE users.

## Funding

VEGA grant [2/0188/13] from the Slovak Academy of Sciences.
